# Do glucocorticoids predict fitness? Linking environmental conditions, corticosterone and reproductive success in the blue tit, *Cyanistes caeruleus*

**DOI:** 10.1098/rsos.170875

**Published:** 2017-10-18

**Authors:** L. J. Henderson, N. P. Evans, B. J. Heidinger, K. A. Herborn, K. E. Arnold

**Affiliations:** 1 Centre for Behaviour and Evolution, IoN, Newcastle University, Henry Wellcome Building, Framlington Place, Newcastle NE2 4HH, UK; 2 Institute of Biodiversity, Animal Health and Comparative Medicine, College of Medical, Veterinary & Life Sciences, The University of Glasgow, Glasgow G12 8QQ, UK; 3 Department of Biological Sciences, North Dakota State University, Fargo, ND 58108, USA; 4 Environment Department, University of York, York YO10 5NG, UK

**Keywords:** avian, evolutionary endocrinology, hormone, stress, woodland ecology

## Abstract

Glucocorticoids, including corticosterone (CORT), have been suggested to provide a physiological link between ecological conditions and fitness. Specifically, CORT, which is elevated in response to harsh conditions, is predicted to be correlated with reduced fitness. Yet, empirical studies show that CORT can be non-significantly, positively and negatively linked with fitness. Divergent environmental conditions between years or study systems may influence whether CORT is linked to fitness. To test this, we monitored free-living blue tits (*Cyanistes caeruleus*) during breeding over 3 years. We quantified foraging conditions during brood rearing, and examined whether they were correlated with parental baseline CORT and reproductive success. We then tested whether CORT predicted fitness. Elevated parental CORT was associated with lower temperatures, greater rainfall and lower territory-scale oak density. Whereas asynchrony with the caterpillar food peak was correlated with reduced nestling mass and fledging success, but not parental CORT. Only low temperatures were associated with both reduced nestling mass and elevated parental CORT. Despite this, parents with elevated CORT had lighter offspring in all years. Contrarily, in 2009 parental CORT was positively correlated with the number fledged. The absence of a direct link between the foraging conditions that reduce nestling quality and elevate parental CORT suggests that parental CORT may provide a holistic measure of conditions where parents are working harder to meet the demands of developing young. As the positive correlation between parental CORT and fledging success differed between years, this suggests that contrasting conditions between years can influence correlations between parental CORT and fitness. Ultimately, as CORT concentrations are intrinsically variable and linked to the prevalent conditions, studies that incorporate environmental harshness will improve our understanding of evolutionary endocrinology.

## 1. Introduction

Glucocorticoids (GCs) are steroid hormones that play a fundamental role in maintaining homeostasis and energy balance [1–4]. GCs are elevated in response to a range of energetically challenging conditions, including food shortage, poor habitat quality and inclement weather conditions [3,5–8]. As similar harsh environmental conditions are linked to a decline in fitness proxies, including reproductive success [9–11], elevated GCs have been suggested to provide a physiological link between challenging environmental conditions and reduced reproductive success [12,13]. Consequently, elevated GCs within individuals and populations are frequently considered to be an indicator of lower relative fitness [12], especially within the context of conservation biology [12–14]. However, a review of the empirical evidence demonstrates that corticosterone (CORT) the main GC in birds can be non-significantly, positively and negatively linked with fitness proxies [12,13,15–17]. Therefore, whether inter-individual variation in CORT provides a physiological link between environmental conditions and reproductive success, and whether CORT titres can be employed to infer individual fitness remain outstanding questions in evolutionary endocrinology.

There is accumulating evidence that the relationship between CORT and fitness is context-dependent. For example, the direction of the correlation between CORT and fitness can vary between populations [18,19], years [20,21], life-history stages [16,17], sexes [15] and reproductive strategies [22]. As baseline CORT is intrinsically linked to an individual's energetic state, variation in the prevalent conditions experienced during breeding has the potential to influence the relationships between parental CORT and reproductive success [12,13,23,24]. Therefore, to improve our understanding of how inter-individual variation in CORT titres vary with fitness proxies, further studies that quantify environmental conditions, parental CORT and reproductive success are required [24]. To date few studies have simultaneously quantified the ecological conditions experienced during breeding, parental CORT and reproductive success to investigate whether elevated CORT links challenging conditions with reduced reproductive success (but see [25,26]). A notable exception to this are a series of studies conducted in seabirds [5,20,21] (for a recent meta-analysis see [27]). These studies provide evidence that low food availability during breeding is consistently associated with both elevated parental baseline CORT and reduced reproductive success. Furthermore, elevated parental CORT was often directly correlated with reduced reproductive success and adult survival [2,3,5,28]. These studies, however, are predominately from seabird colonies that have experienced significant population declines caused by abrupt drops in prey abundance [2,3,5,28]. Therefore, it is unclear whether similar results will be evident in stable populations, which have not experienced similar deterioration of their breeding environment.

Under mild fluctuations in environmental conditions, like a minor reduction in food availability, parents may compensate through behavioural flexibility [29–31]. Functionally, the elevation of baseline CORT in response to environmental challenges can mobilize fat reserves for energetically demanding behaviours [32]. In birds, elevated baseline CORT has been associated with increased foraging duration [33] and nestling provisioning rates [34,35]. Therefore, parental CORT elevation is predicted to promote behaviours that prevent a mild decline in conditions from adversely affecting offspring survival [36]. In this case, low food availability may be associated with elevated parental CORT, but not result in a negative correlation between parental CORT and offspring number [14,29]. However, while a mild decline in environmental conditions during rearing may not influence offspring number, it could reduce nestling condition [37], resulting in a negative correlation between parental CORT and nestling quality. This would have important consequences for individual fitness, as fledging mass can influence future survival and reproductive success of offspring [38–41]. Therefore, when investigated whether parental CORT predicts reproductive success, it is important to assess multiple measures of breeding success [42].

Additionally under relatively benign conditions, rearing offspring may present the greatest challenge experienced by parents, and consequently elevated CORT concentrations may facilitate offspring care [12]. In agreement with this, a number of studies provide evidence that parental CORT concentrations can be positively correlated with offspring number during rearing [16–18,43–45]. There is also evidence that a positive correlation between parental CORT and breeding success can be context-dependent [12,18,24]. For example, between two geographically distinct populations of blue tit (*Cyanistes caeruleus*), a positive correlation between brood size and baseline CORT was only evident in the population that had comparatively higher ectoparasite loads [18]. Importantly, we would predict a positive correlation between parental CORT and brood size, only when parents with larger broods experience disproportionately high energetic demands but are still able to successfully raise their broods to fledging.

To investigate whether variation in the prevalent conditions experienced during breeding has the potential to influence the relationships between parental CORT and fitness proxies, a free-living population of blue tits were studied over 3 years that differed in environmental conditions. The blue tit provides an ideal model system, as the ecological variables that influence fitness are well known, and ecological conditions often vary significantly between years creating a natural experiment [46–48]. To quantify the foraging conditions experienced during brood rearing, we measured asynchrony between breeding and the peak in caterpillar abundance, weather variables and territory-scale oak density (inferred from the distance to the nearest oak from each nest), all of which can influence both reproductive success and the energetic demands of breeding birds during brood rearing [37,49–51]. We investigated whether, (i) inclement foraging conditions were associated with elevated baseline CORT; (ii) the same inclement foraging conditions were correlated with reduced reproductive success, i.e. fledging number and nestling mass; and (iii) variation in parental baseline CORT predicted reproductive success.

## 2. Material and methods

### 2.1. Field site and fitness proxies

Blue tits breeding in nest-boxes in oak-dominated woodland around Loch Lomond, Scotland (56°13′ N, 4°13′ W) were studied for 3 years from April to June 2008–2010. Nest-boxes were monitored regularly from the onset of nest building to establish the first date an egg was laid (lay date), clutch size and hatching date (*n* = 2008: 144, 2009: 83, 2010: 50). When more than 50% eggs had hatched, this was considered day 1. To measure nestling condition, on day 14 after hatching all nestlings were weighed to the nearest 0.05 g with a Pesola spring scale. To establish fledging number, nest-boxes were visited after fledging to check for any unfledged offspring. There was no evidence of predation at the nests included in this study. The smaller number of nests in 2009 and 2010 was because more than 50 nests were used in a manipulative study [52].

### 2.2. Blood sampling

To control for effects of breeding stage, parental baseline CORT was measured at the same point during brood rearing across all years. Birds were captured on their nest, during provisioning on day 5–7 after chicks hatched. A small blood sample was obtained (about 80–100 µl) with the aid of a standard heparinized capillary tube after puncture of the brachial vein with a 25 gauge needle. All blood samples were collected within 3 min of the initial blockage of the nest-box entrance [53]. CORT samples were considered to be baseline as the time spent by researchers at the nest before capture, time between sampling and initial disturbance of the nest and time of day were not related to CORT (*n* = 113; duration at nest-box, *t* = 0.43; *p* = 0.67; sampling time, *t* = 0.80, *p* = 0.43; and time of day, *t* = −1.41, *p* = 0.16). Parents were sexed based on the presence or absence of a brood patch [54].

### 2.3. Foraging conditions

**Figure 1.**
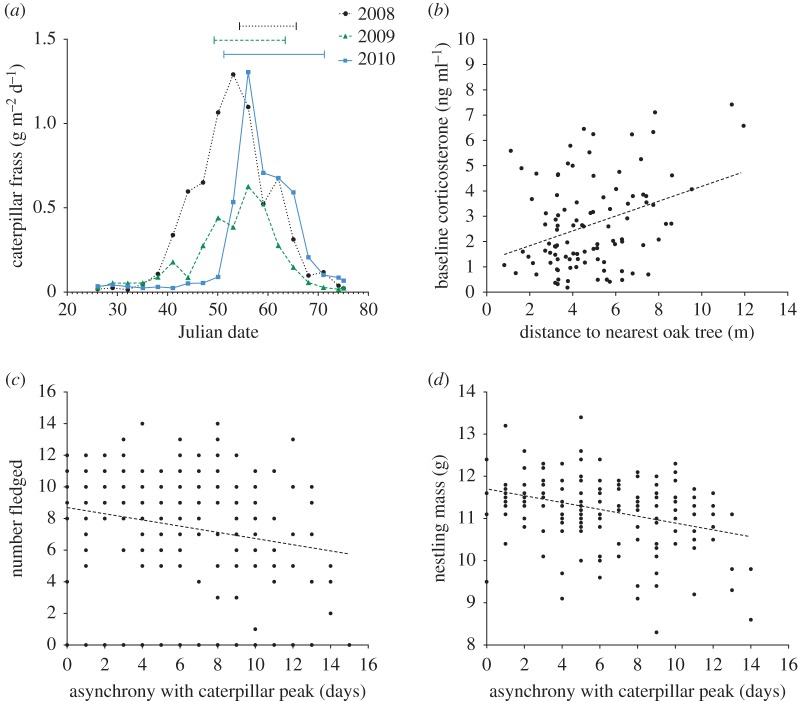
(*a*) Caterpillar abundance measured by frass fall collection (g m^−2^ day^−1^) in April to June 2008–2010 (Julian date, 1 April = 0). Horizontal lines indicate the period when blue tit nestlings were 10 days old in each year. (*b*) Distance to the nearest oak tree was positively correlated with parental baseline CORT in all years (greater distance from the nest indicates a territory with fewer oak trees), (*c*) asynchrony with the caterpillar food peak was negatively correlated with number fledged and (*d*) nestling mass in all years.

The foraging conditions experienced by breeding birds were assessed in three ways. (i) The asynchrony between breeding birds and the peak in caterpillar abundance was estimated by the collection of frass from April to June each year. Asynchrony rather than absolute frass fall was used to assess food availability, as it provides a higher resolution assessment of food availability for individual nests within years, and is more robust to variation between trees, and rainfall between years ([55–58]; see also electronic supplementary materials, S1). For example, 2009 had significantly higher rainfall than the other years, and lower absolute frass fall (figure 1*a*). Owing to this, it is not possible to say whether there were fewer caterpillars, or if the heavier rainfall dissolved a greater proportion of the frass. As the woodland is oak-dominated (more than 95% trees), and caterpillars are at their highest densities in oak foliage [11,59,60], frass fall was collected from 20 mature oak trees and assessed by measurement of the dry weight. To calculate the asynchrony between breeding birds and the peak in caterpillar abundance, the number of days between the date of maximum frass abundance (mean calculated from all trees) and the date when nestlings were 10 days old was calculated for each nest (figure 1*a*). At 10 days of age, nestlings are growing at their fastest rate, so their nutritional requirements are at their highest [61]. To allow for comparisons between years, dates were converted to Julian with 0 = 1 April. For the full methodology, see electronic supplementary materials, S1. (ii) Territory-scale oak density was assessed by measuring the distance (m) between each nest and the nearest oak tree. This time-efficient method was used because, in agreement with previous studies [62], we found for a subset of territories that the number of oak trees within a 25 m radius of the nest was negatively correlated with the distance to the nearest oak (Pearson's correlation; *r* = −0.94, *n* = 20, *p *< 0.01). (iii) Weather conditions were assessed using data collected at a meteorological station less than 10 miles from the field site. Total rainfall (mm) and max temperature (°C) were collected every 24 h throughout the breeding season for all years. To assess the impact of prolonged weather conditions upon CORT, the mean rainfall and max temperatures experienced 72 h following blood sample collection were calculated for each individual [63]. Weather conditions 24–48 h prior to blood sampling were not related to parental CORT. To investigate the impact of weather conditions upon fitness proxies, the mean rainfall and max temperatures experienced during the majority of chick development, i.e. day 4–14 after hatching, were calculated for each nest.

### 2.4. Hormone assays

Corticosterone was extracted from 5–20 µl aliquots of plasma using diethyl ether and concentrations measured using a double antibody radioimmunoassay [64]: primary antibody (Esoterix B183), secondary antibody (Sigma goat anti-rabbit) and [3H]-corticosterone label (GE Healthcare, UK). The extraction efficiency was 85–100%. Recoveries were measured for each sample to allow calculation of individual plasma CORT concentrations. Pooled plasma samples were used in all assays as standards to assess inter-assay variation. The detection limit of the assays (*n* = 3) averaged 0.03 ng/ml and the intra- and inter-assay coefficients of variation were 9 ± 6% and 10 ± 4%, respectively.

### 2.5. Statistical analysis

To investigate how foraging conditions differed between years, Kruskal–Wallis tests were employed, as transformations could not normalize the distribution or variance of the data. A GLM with a binomial error structure was used to compare the number of days it rained between years (hereafter referred to as rain days). All CORT data were square root transformed because of non-normality.

To assess the influence of foraging conditions upon parental CORT, GLMs were used. Two-way interactions between foraging conditions, sex and year were fitted. Only one parent was used from each nest, and when parents were captured in more than 1 year, they were only used in the analysis in the first year they were caught. This was to avoid pseudo-replication, and because we captured few pairs (*n *< 4), and captured few birds in more than 1 year (*n *< 6). There was evidence of autocorrelation in some of the foraging conditions. Maximum temperature and rainfall were significantly negatively correlated (Spearman's rho: *r* = −0.38, *n* = 150, *p *< 0.001). Therefore, models were run twice, once with rainfall and once with temperature. As rainfall was run in separate models, if rainfall was significant, it is reported in the results section; models that included temperature are reported in tables. Lay date was not included in these models as it was not correlated with parental CORT (GLM *t* = −0.11, *p* = 0.91), but was correlated with asynchrony with the caterpillar peak (GLM *t* = 6.15, *p *< 0.01).

Lay date was a significant predictor of brood size (GLM *t* = −4.89, *p *< 0.01) and asynchrony with the caterpillar peak (GLM *t* = 6.15, *p *< 0.01). Therefore, to control for this, we used the residuals of a linear regression between number fledged or nestling mass and lay date, as the dependent variables in the GLMs investigating whether parental CORT or foraging conditions explained variation in the breeding success. Not all parents were sampled for baseline CORT, so models investigating number fledged and nestling mass have a larger sample size than those investigating parental CORT. Also we were not able to measure nestling mass in all nests where we captured parents for baseline CORT, therefore nestling mass models have a smaller sample size than number fledged.

Models were optimized using backward elimination of non-significant terms (*p *> 0.05), and models were compared using ANOVA. Terms remained in the model if their deletion caused a significant increase in deviance (*p *< 0.05) [65]. Models were validated to verify that underlying statistical assumptions were not violated; normality was assessed by plotting theoretical quantiles versus standardized residuals (quantile–quantile plots); homogeneity of variance was evaluated by plotting residuals versus fitted values, and nonlinearity was evaluated by plotting residuals versus explanatory variables [65]. All statistical analyses were conducted using R v. 2.12.2 [66].

## 3. Results

### 3.1. Foraging conditions

In 2008 and 2010, birds were more asynchronous with the peak in caterpillar abundance than 2009 (table 1, figure 1*a*, *H* = 45.85, *p *< 0.001). In 2009, there were significantly more rain days than in the other 2 years of the study (table 1, *t* = 2.16, *p* = 0.03); and in 2008, maximum temperatures were higher than in the other 2 years (table 1, *H* = 6.29, *p* = 0.04). Despite differences in the nest-boxes used by birds between years, our proxy of territory-scale oak density, the distance from the nest to the nearest oak tree, did not differ between years (*H* = 2.39, *p* = 0.30).

**Table 1.** Inter-annual variation in asynchrony between breeding birds and the caterpillar food peak, rainfall, max temperature, parental baseline CORT, number fledged and nestling mass for free-living blue tits (2008–2010). Mean ± s.e. are shown; values in italics differ significantly from the other years; * denotes significance at *p* < 0.05; and ** denotes significance at *p* < 0.001.

	2008	2009	2010
asynchrony with caterpillar peak (days)	8.28 ± 0.25	2.68 ± 0.24**	5.28 ± 0.40**
rain days (%)	40	62*	50
temperature (°C)	17.39 ± 0.39*	16.16 ± 0.42	16.42 ± 0.47
adult CORT (ng ml^−1^)	2.93 ± 0.25	3.14 ± 0.25	1.02 ± 0.15**
number fledged	7.38 ± 0.34	7.16 ± 0.50**	8.20 ± 0.46
nestling mass (g)	11.05 ± 0.10	11.34 ± 0.09	11.39 ± 0.12

### 3.2. Corticosterone and foraging conditions

Temperature was negatively correlated with parental CORT (table 2*a*), and rainfall was positively correlated with parental CORT in all years (GLM *t* = 2.09, *p* = 0.04). Parents breeding in territories with fewer oak trees had higher baseline CORT than those breeding in oak-dense territories (table 2*a*, figure 1*b*). However, asynchrony with the caterpillar peak was not correlated with parental CORT. Baseline CORT was significantly lower in 2010 compared to the other years (tables 1 and 2*a*), and CORT concentrations were significantly lower in males compared with females (table 2*a*).

**Table 2.** The results of GLMs investigating whether parental sex, year or foraging conditions were correlated with (*a*) parental CORT (*n* = 113), (*b*) number fledged (*n* = 258) and (*c*) nestling condition (*n* = 164). Values in italics denote statistically significant factors.

factor	d.f. effect	s.e.	*t*	*p*
(*a*) baseline CORT				
year	2	1.12	−5.33	*<0*.*001*
sex	1	0.10	−3.45	*<0*.*001*
oak density	1	0.02	2.92	*0*.*004*
temperature	1	0.03	−2.00	*0*.*048*
(*b*) number fledged				
year	2	0.33	−4.35	*<0*.*001*
asynchrony	1	0.04	−2.90	*0*.*003*
(*c*) nestling mass				
year	2	0.21	−1.11	0.268
asynchrony	1	0.02	−3.31	*0*.*001*
temperature	2	0.06	2.08	*0*.*039*

### 3.3. Reproductive success and foraging conditions

**Figure 2.**
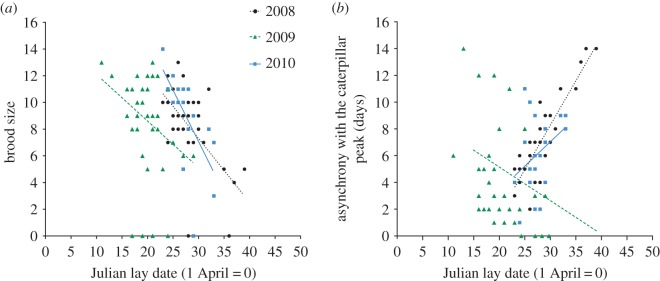
(*a*) Earlier laying birds had larger brood sizes in all years. (*b*) Earlier laying birds were more synchronous with the caterpillar peak in 2008 and 2010, but this pattern was reversed in 2009 as earlier laying birds were less synchronous with the caterpillar peak.

In all years, nests that were more asynchronous with the caterpillar peak fledged fewer offspring (figure 1*c*, table 2*b*) and contained lighter nestlings (figure 1*d*, table 2*c*). Temperature was positively correlated with nestling mass in all years (table 2*c*). Rainfall and territory-scale oak density was unrelated to number fledged and nestling mass. Nestling mass did not differ between years, but the number fledged was lower in 2009 (tables 1 and 2*b*). In all years, parents with earlier lay dates had larger broods (figure 2*a*, GLM *t* = −4.89, *p *< 0.01). In 2008 and 2010, parents with earlier lay dates were also more synchronous with the caterpillar peak (figure 2*b*). But this was reversed in 2009, as parents with earlier lay dates were less synchronous with the caterpillar peak (figure 2*b*, GLM Year × Asynchrony: *t* = −6.31, *p *< 0.01).

### 3.4. Corticosterone and fitness proxies

**Figure 3.**
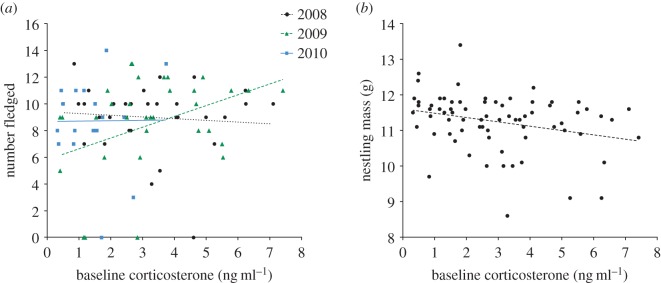
(*a*) There was a year-specific relationship between parental CORT and number fledged, with CORT positively correlated with number fledged in 2009 only. (*b*) Parental CORT was negatively correlated with nestling mass in all years.

The relationship between parental CORT and number fledged varied between years, shown by the significant effect of the interaction ‘CORT × Year’ on the number of chicks fledged (table 3*a*). This was driven by a significant positive correlation between CORT and number of chicks fledged in 2009 only (figure 3*a*, 2009: GLM *t* = 2.20, *p* = 0.03). Nestling mass was negatively correlated with parental CORT in all years (table 3*b*, figure 3*b*).

**Table 3.** The results of GLMs investigating whether variation in (*a*) number of fledglings (*n* = 113) or (*b*) nestling mass (*n* = 86) was explained by year, parental CORT or the interaction between parental CORT and year. Values in italics denote statistically significant factors.

factor	d.f. effect	s.e.	*t*	*p*
(*a*) number fledged				
year	2	0.92	−2.51	0.014
CORT	1	0.38	−0.77	0.445
year × CORT	2	0.53	2.11	*0.037*
(*b*) nestling mass				
CORT	1	0.14	−2.60	*0.011*

## 4. Discussion

Our results add to evidence that baseline CORT is elevated in response to energetically challenging conditions in free-living birds [6,63,67,68]. Prodigious rainfall, cold temperatures and fewer oak trees within a territory were correlated with elevated parental CORT. However, over the 3 years of our study the foraging conditions that elevated parental CORT did not consistently predict reproductive success. Only low temperatures were associated with both reduced nestling mass and elevated parental CORT. Furthermore, asynchrony with the caterpillar peak strongly predicted nestling mass and number fledged, but was not correlated with parental CORT. Importantly, the variation in foraging conditions within and between years was mild, demonstrated by the high breeding success across years. Under mild variation in environmental conditions, we may not predict a negative correlation between parental CORT and offspring survival. This is because parents can increase provisioning and brooding behaviours in response to a mild decline in food abundance or weather conditions [3,49], which in turn can be facilitated by elevated baseline CORT [33–35].

Yet, parental CORT was negatively correlated with nestling mass in all years. As parental CORT was unrelated to nestling mass on day 4 after hatching (GLM *t* = −1.29, *p* = 0.20), our results do not provide evidence that parents with elevated CORT laid lighter offspring. Rather this result suggests that, in breeding blue tits, parental CORT provides a physiological link between conditions during rearing and nestling quality close to fledging. Hence, parental CORT may provide a holistic measure of how challenging parents perceive their environment, and be indicative of conditions where parents are unable to provide adequate resources for their young [3,19,33]. In female tree swallows (*Tachycineta bicolor*), experimentally reducing flight efficiency during provisioning was associated with both elevated baseline CORT and reduced nestling provisioning rates [26]. Our results highlight the importance of measuring multiple measures of breeding success, when investigating whether CORT titres can be employed to infer individual fitness. Ultimately, our results show that hormonal measures can be consistently linked to nestling quality across multiple years that differ in foraging conditions.

Parental CORT was positively correlated with number of chicks fledged in one year. This result supports previous studies that show that parental CORT can be positively correlated with reproductive success, and therefore not consistently linked to a decline in fitness proxies [18,43–45]. As this relationship differed between years, this suggests that contrasting environmental conditions between years influenced the correlations between parental CORT and fitness. In our study population, parents with earlier lay dates had larger broods. In addition, in 2008 and 2010 parents with earlier lay dates were also more synchronous with the caterpillar peak. But this was reversed in 2009, as parents with earlier lay dates were less synchronous with the caterpillar peak. In blue tits, the degree of mismatch between peak caterpillar abundance and chick demand can greatly increase parental foraging costs [9,49,69]. Therefore, while parents were overall more synchronous with the caterpillar peak in 2009; the mismatch between chick demand and caterpillar abundance for parents with larger broods, probably resulted in greater foraging effort. Additionally, in 2009 there was significantly more rainfall, and fledging success was lower compared to the other years of the study. This suggests that foraging conditions were more challenging in this year.

Overall, our results show that a positive correlation between brood size and parental CORT can occur when parents with larger broods experience disproportionately high energetic demands, but are still able to successfully raise their broods to fledging. In addition, birds with earlier lay dates are probably superior-quality individuals [58,61], capable of increasing workloads to provide for young [16,44]. For example, in tree swallows, females that had greater increases in baseline CORT in response to brood enlargement were able to provision their offspring at a higher rate [44]. Our results provide evidence that measuring the environmental conditions experienced by breeding birds will more accurately predict the context dependence of correlations between parental CORT and fitness. Furthermore, experimental studies that manipulate ecological conditions, within a biologically relevant range during brood rearing, would provide causal evidence that the prevalent conditions can influence the relationship between CORT titres and fitness proxies.

Previous studies have suggested that a positive correlation between parental CORT and fledging number indicates that individuals with elevated CORT have higher relative fitness [12,17]. However, there may be costs associated with elevating CORT in response to brood rearing that may trade off against future reproduction and survival [28,70,71]. For example, in giant petrels (*Macronectes* spp.) mothers with higher CORT during breeding were more likely to defer breeding in the following year [72]. The context dependence of the correlation between parental CORT and fledging number, and the negative correlation between parental CORT and nestling mass in our study suggests that a positive correlation between CORT and reproductive success in a single year may not be predictive of lifetime reproductive success. Furthermore, while there was positive correlation between CORT and number fledged in 2009, parents with elevated baseline CORT also fledged lighter offspring. As mass at fledging is predictive of future survival and reproductive success of offspring [38–41], parents with elevated CORT in 2009 may have suffered reduced reproductive success in that year. Overall, studies that incorporate the concepts of reproductive trade-offs and reproductive strategies would be insightful (e.g. [72–75]).

## 5. Conclusion

This study shows that circulating parental CORT concentrations can be indicative of offspring quality. In addition, the year-specific positive correlation between parental CORT and number fledged suggests that variation in conditions between years may alter the relationship between parental CORT and fitness proxies. Our results highlight the importance of measuring multiple measures of reproductive success when trying to establish whether inter-individual variation in CORT predicts fitness. Ultimately, as CORT concentrations are intrinsically variable and linked to the prevalent conditions, further studies that incorporate environmental harshness will improve our understanding of evolutionary endocrinology.

## Supplementary Material

Supplemental Information S1

## Data accessibility

Data are available to download from Dryad: (http://dx.doi.org/10.5061/dryad.j0475) [76].
