# Supplementary material for: Do glucocorticoids predict fitness? Linking environmental conditions, corticosterone and reproductive success in the blue tit, Cyanistes caeruleus
Source: R Soc Open Sci. 2017 Oct 18;4(10):170875. doi: 10.1098/rsos.170875 (PMC5666270; doi:10.1098/rsos.170875)
Supplement: Supplemental Information S1 [file rsos170875supp1.docx]

**Royal Society Open Science**

**Do glucocorticoids predict fitness? Linking environmental conditions, corticosterone and reproductive success in the blue tit, *Cyanistes caeruleus***

L. J. Henderson*^a, b^, N. P. Evans^b^, B. J. Heidinger ^b, c^ K. A. Herborn^a, b^ and K. E. Arnold ^b, d^

^a^ Centre for Behaviour and Evolution, IoN, Newcastle University, Henry Wellcome Building, Framlington Place, Newcastle NE2 4HH, UK

^b^ Institute of Biodiversity, Animal Health and Comparative Medicine, College of Medical, Veterinary & Life Sciences, The University of Glasgow, Glasgow, G12 8QQ, UK

^c^ Department of Biological Sciences, North Dakota State University, Fargo, ND 58108

^d^ Environment Department, University of York, YORK, YO10 5NG, UK

**Supplementary Materials S1**

### CATERPILLAR ABUNDANCE

To assess relative caterpillar abundance throughout the breeding period, caterpillar frass fall was collected from April to June each year using standard methods (*1*–*4*). Caterpillars are at their highest densities in oak foliage, therefore, frass fall was measured from 20 mature oak trees (circumference: 1.2-1.7 m). To take account of any variation across the field site, trees selected for frass collection were distributed throughout the woodland (> 500m apart). Frass nets were 0.5 m^2^ and made of cotton. Two nets were placed underneath each tree, 1 m from the trunk on the east and west side. Frass nets were emptied every 3 days unless rain prevented collection, in which case they were emptied once they were dry or on the next day frass was collected. Samples were then stored at -20˚C prior to analysis. For each frass sample, larger debris such as leaves and bark were separated from frass using a medium sieve (mesh size 1.4mm). To obtain dry mass, the remaining samples were placed in a convection oven and dried for 48 hours at 60˚C. Samples were then stored in a dessicator to avoid absorption of moisture as samples cooled. Samples were sieved again (mesh size 0.7mm) to separate frass from other small pieces of debris. Frass pellets from each sample were then weighed to the nearest mg. When heavy rain had dissolved the frass pellets and reduced them to a powder, this material was weighed and included in the total weight of the frass. Asynchrony with the caterpillar peak was employed as a measure of food availability, rather than absolute frass fall for two reasons; i) asynchrony provides a more accurate assessment of available food, because breeding pairs gather insects from multiple trees within their territory and individual trees varied up to 10-fold in absolute frass fall. However, the date trees showed peak frass fall was consistent, >90% trees showed peak frass fall on the same collection date. ii) The study site is in a temperate rain forest and can experience high rainfall. Absolute frass fall measured by open nets can be affected by rainfall, as it can dissolve collected frass (*5*). This can cause comparisons between years of absolute frass fall to be inaccurate, when years differ in rainfall. For example, 2009 had significantly higher rainfall than the other years, and lower absolute frass fall (figure 1a). Due to this it is impossible to say whether there were fewer caterpillars, or if the heavier rainfall dissolved a greater proportion of the frass. However, the peak date of frass fall should be more resilient to differences in rainfall between years. Therefore, asynchrony between peak frass fall and the most demanding stage of chick rearing provides a more accurate representation of available food, that is resilient to variation between trees and weather conditions between years.

**References**

1. J. Blondel *et al.*, A Thirty-Year Study of Phenotypic and Genetic Variation of Blue Tits in Mediterranean Habitat Mosaics. *Bioscience*. **56**, 661–673 (2006).

2. I. Tremblay, D. W. Thomas, M. M. Lambrechts, J. Blondel, P. Perret, Variation in blue tit breeding performance across gradients in habitat richness. *Ecology*. **84**, 3033–3043 (2003).

3. M. E. Visser, L. J. M. Holleman, P. Gienapp, Shifts in caterpillar biomass phenology due to climate change and its impact on the breeding biology of an insectivorous bird. *Oecologia*. **147**, 164–172 (2005).

4. M. Fischbacher, B. Naef-Daenzer, L. Naef-Daenzer, Estimating caterpillar density on trees by collection of frass droppings. *Ardea*. **86**, 121–129 (1998).

5. N. Kamata, Y. Igarashi, Problems in estimating the density of larval beech caterpillar, Quadricalcarifera punctatella (Motschulsky) (Lep., Notodontidae), using frass drops by modification of the Southwood-Jepson method. *J. Appl. Entomol.* **118**, 92–99 (1994).
